# Short-term antagonism between bacteriophages and macrophages decreases with bacteria-phage coevolution

**DOI:** 10.1093/ismejo/wrag116

**Published:** 2026-05-08

**Authors:** Meaghan Castledine, Zuzanna Szczutkowska, Andrew Matthews, Sarah K Walsh, Rai Lewis, Suzanne Kay, Janet A Willment, Gordon D Brown, Angus Buckling

**Affiliations:** Department of Ecology & Conservation, Environment and Sustainability Institute, University of Exeter, Penryn, Cornwall, TR10 9EZ, United Kingdom; Department of Ecology & Conservation, Environment and Sustainability Institute, University of Exeter, Penryn, Cornwall, TR10 9EZ, United Kingdom; Department of Ecology & Conservation, Environment and Sustainability Institute, University of Exeter, Penryn, Cornwall, TR10 9EZ, United Kingdom; Medical Research Council-University of Glasgow Centre for Virus Research, Sir Michael Stocker Building, Garscube Campus, 464 Bearsden Road, Glasgow, G61 1QH, Scotland; Department of Ecology & Conservation, Environment and Sustainability Institute, University of Exeter, Penryn, Cornwall, TR10 9EZ, United Kingdom; Department of Ecology & Conservation, Environment and Sustainability Institute, University of Exeter, Penryn, Cornwall, TR10 9EZ, United Kingdom; Department of Biosciences, Faculty of Health and Life Sciences, Medical Research Council Centre for Medical Mycology at the University of Exeter, Exeter, EX4 4QJ, United Kingdom; Department of Biosciences, Faculty of Health and Life Sciences, Medical Research Council Centre for Medical Mycology at the University of Exeter, Exeter, EX4 4QJ, United Kingdom; Department of Ecology & Conservation, Environment and Sustainability Institute, University of Exeter, Penryn, Cornwall, TR10 9EZ, United Kingdom

**Keywords:** phage therapy, macrophages, immune system, *Pseudomonas aeruginosa*, experimental evolution, bacteriophages

## Abstract

Phage therapy, the use of viruses that infect bacteria (bacteriophages), is a promising complement to antibiotics during the antimicrobial resistance crisis, but treatment success is very variable. A key variable, which likely influences treatment outcomes, is how different immune components interact with bacteriophage, with studies finding neutrophils work synergistically while macrophages work antagonistically with bacteriophage. However, many of these studies characterize interactions and outcomes over short timescales, not considering the potential for the evolution of resistance to bacteriophages which can itself greatly affect treatment outcomes. Here, we measure how macrophages and bacteriophages affect densities and resistance evolution of the pathogen *Pseudomonas aeruginosa* in vitro. Consistent with previous studies, we find macrophages interact antagonistically with bacteriophages in the short term. However, this antagonism was lost following bacterial population recovery associated with rapidly evolved resistance to bacteriophages. Macrophages resulted in greater net levels of resistance and hindered increases in bacteriophage infectivity, but this did not lead to differences in bacteria-phage population dynamics. This work emphasizes the importance of characterizing the effect of the immune system on phage therapy outcomes over both shorter- and longer- timescales.

## Introduction

In 2019, 4.95 million deaths were associated with antimicrobial resistant (AMR) infections, with this figure expected to double by 2050 [1]. To combat the AMR crisis, new treatments are required such as phage therapy [2]. Phage therapy uses viruses (bacteriophages) that specifically infect and kill bacteria to treat bacterial infections with reported success in patients and animal models [2–4]. As bacteriophages are typically species-specific and self-amplifying at infection sites, they can target pathogenic bacteria without disturbing microbiomes [5, 6]. However, phage therapy outcomes are highly diverse and clinical trials have produced mixed results for determining efficacy in pathogen eradication [2, 7, 8].

The immune system is likely to be responsible for much of this unpredictability with patients varying in immunocompetence and different pathogens varying in their ability to evade immune components. Although the immune system is often assumed to be key for phage therapy success [9, 10], experimental work has yielded mixed-results with immune components both aiding and hindering phage therapy success [3, 11, 12]. For example, mice that can produce neutrophils had significantly higher survival probabilities than immuno-compromised mice when treated for an infection with phage therapy [13]. In contrast, macrophages can reduce phage efficacy by internalizing phages, therefore resulting in poorer treatment outcomes in mice [14].

These interactions between immune components, bacteriophage, and bacteria are further complicated by phage resistance. Bacterial pathogens can rapidly evolve resistance to bacteriophages [15], and bacteriophages can in turn evolve to overcome resistance [16, 17]. Recent theory suggests that synergy with the immune system and bacteriophages may be contingent on (co)evolution occurring, with immune cells clearing resistant bacteria and viruses clearing susceptible populations [13, 18]. Experimental studies report bacteriophage resistance to be associated with reduced bacterial survival in insect haemolymph [19] and increased phagocytosis [20]; although, phage resistance can also positively covary with resistance to phagocytosis [11]. These different outcomes may in part be explained by the types of resistance mutation [21]. For example, biofilm production is a common mechanism of bacteriophage resistance [22, 23], that can also confer resistance to phagocytes, and increase release of pro-inflammatory cytokines [21]. Additionally, bacteriophages can be internalized by phagocytes [14, 24] (or bound by antibodies [25]). Synergy between the immune system and bacteriophages will then be dependent on the relative rates of replication and phagocytosis between pathogens and viruses, which will be affected by phage resistance [3].

**Figure 1:**
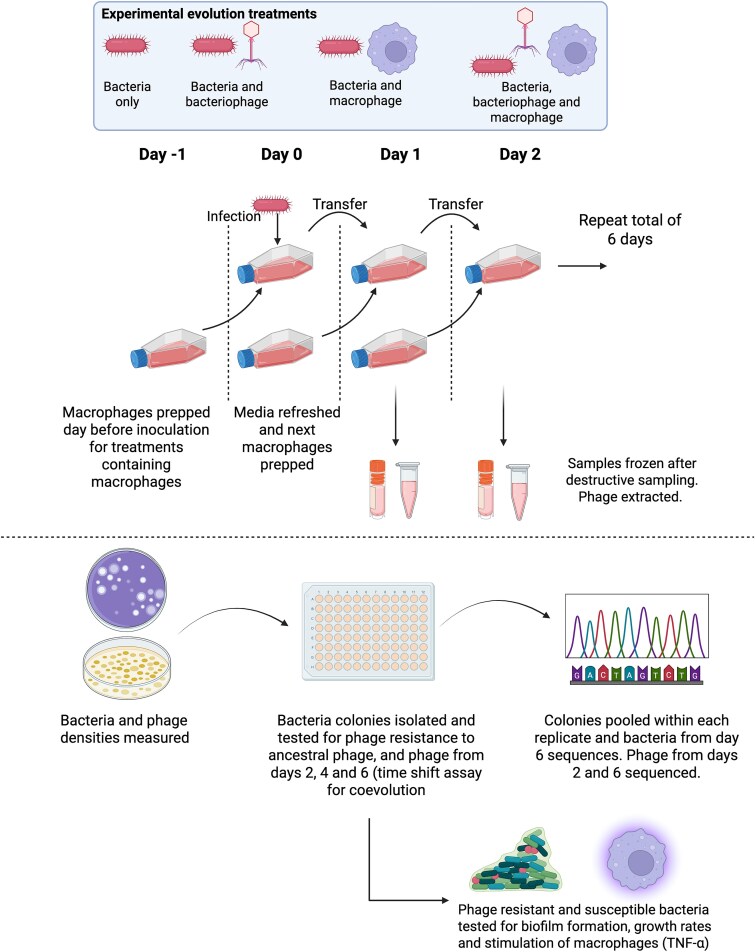
Overview of experimental design for experimental evolution with bacteria, bacteriophages, and macrophages. For treatments containing macrophages, macrophages were seeded the day before inoculation/transfer. Bacteria and bacteriophage samples were taken daily to measure changes in bacteria and bacteriophage density. After experimental evolution, cultures were plated to isolate colonies, which were tested for phage resistance in a time-shift assay for measuring coevolution. Isolated colonies were also used for sequencing, biofilm, growth rate and macrophage stimulation analysis. Created in BioRender. Castledine, M. (2026) https://BioRender.com/2f6r8n9.

Conflicting findings on the role of the immune system in phage therapy, suggest that the interactions between bacteria, bacteriophage and immune system components could significantly change through time as a function of bacteria-phage (co)evolution. We test this hypothesis using an in vitro system of macrophages, therapeutic bacteriophages (14–1 and PNM) and the pathogenic bacterium *P. aeruginosa* (mucoid strain P573). Macrophages also retain high cell viability during in vitro assays and are one of the first cell types to respond during an infection [26], thereby making these cells an attractive and applicable model system for understanding longer term interactions in phage therapy. To this end, we passaged *P. aeruginosa* through daily cultures of macrophages (RAW 264.7) and examined changes in bacteria and bacteriophage population dynamics, and characterized subsequent evolutionary and phenotypic changes (Fig. 1).

## Materials and methods

### Cell line

Macrophages, RAW 264.7 cell lines (8 passages; lot number 70041657) were purchased from American Type Culture Collection (ATCC® TIB-71). Culture medium consisted of DMEM (Dulbecco’s Modified Eagle's Medium, GlutaMAX) supplemented with 10% FBS (foetal bovine serum) and 1% penicillin/streptomycin (for subculturing only, not used in experiments with bacteria or bacteriophages). Cells were incubated at 5% CO_2_ and 90% humidity at 37°C. Cells were passaged at ~70% confluency at a 1:10 seeding ratio in T-75 (75 cm^3^) flasks.

### Bacteria and bacteriophage strains

*P. aeruginosa* strain CN573 = PSE143 is a mucoid isolate which was selected based on its clinical origins [27, 28]. Since *P. aeruginosa* strains can occupy clinical and natural environments, we selected a strain that would have a genetic background relevant for phage therapy research and susceptibility to clinically relevant bacteriophages. CN573 belongs to *P. aeruginosa* phylogroup A and has at least two anti-phage defence systems including CRISPR-cas and restriction modification (type-II) [29]. Another three anti-phage systems were found by running the reference genome through PADLOC (PADLOC_v1.2.0 with PADLOCDB_v1.5.0 [30]), including Gabija, PD-T4–6 and PifA (Table S1). Bacteriophages included 14–1 and PNM, strains that are used together for phage therapy, most notably in the PhagoBurn clinical trial [31]. This bacteriophage cocktail has previously been characterized to ensure no prophage elements are present or that the bacteriophage carry toxin-encoding genes that may increase bacterial resistance and/or virulence [28]. Bacteriophages had been purified of endotoxins to prevent harm to eukaryotic cells (including the macrophages in this study) [28]. As 14–1 infects via a lipopolysaccharide (LPS) [32, 33] receptor and PNM uses type IV pili [34], resistance to one bacteriophage is unlikely to result in cross-resistance and neither bacteriophage should compete for receptor sites [35]. Bacteriophages were purified of endotoxins using Endotraps (Lionex) prior to experimental use. Endotoxins were measured using a Chromogenic Endotoxin Quantification Kit with target endotoxin concentrations <0.5 EU/mL.

We confirmed bacteria interacted with this macrophage line using confocal microscopy. Although there is not a fluorescent marked strain of CN573, it does emit some natural autofluorescence from pyoverdine. Briefly, 3 × 10^5^ macrophages were seeded onto poly-l-lysine and acid washed treated coverslips (Corning BioCoat) in 12-well plates with 500 μL DMEM. Cultures were grown for 4 hours to facilitate adhesion. 3 mL aliquots of overnight bacterial culture were centrifuged at 2.4× g for 5 minutes and washed twice with PBS (phosphate buffered saline). Bacteria were inoculated onto coverslips with a target MOI of 100:1 (bacteria to macrophage) and incubated for 1–2 hours for imaging. Coverslips were removed and gently washed in PBS to remove media and unbound bacteria. Images were acquired using a confocal fluorescence microscope (Leica AF6000) set up as follows: objective×20 plan apochromatic; excitation wavelength 488 nm, emission wavelength 493–547 nm. Processing and analysis of images was conducted using Leica software (LAS X version 3.5.1.18803). Images confirmed the presence of intracellular bacteria (Fig. S1), with no fluorescence detectable in macrophage-only controls (Fig. S2).

### Experimental evolution

For experimental evolution, four treatments were established with six replicates per treatment: bacteria only, bacteria with macrophages, bacteria with bacteriophages, and bacteria with both macrophages and bacteriophages. Macrophages were seeded into T-25 (25 cm^3^) tissue culture flasks with 10^6^ cells in 5 mL tissue culture medium and incubated overnight to reach ~80–90% confluency. Cell culture medium was refreshed prior to inoculation. Overnights of *P. aeruginosa* (grown in Luria Broth (LB) at 37°C, shaking at 180 r.p.m) were diluted to 0.1 OD (0.1 optical density (OD_600_; 600 nm wavelength)) in PBS and 20 μL was inoculated into each microcosm (2 × 10^6^ CFUs; ~1:1 bacteria to macrophage ratio). To bacteriophage cultures, 40 000 PFUs were inoculated (2 μL of bacteriophage stock at 20000 PFUs/uL) to achieve an MOI of 0.02 (bacteria to bacteriophage ratio). Flasks were incubated as above. Serial transfers took place every 24 hrs for six days. Twenty-four hours prior to transfer, fresh macrophages were seeded into flasks mimicking starting conditions with media refreshed. At each transfer, replicates of each evolution line were vortexed and 1 mL of culture was removed and centrifuged at 5000 r.p.m (2000× g) (Progen GenFuge 24D centrifuge) for 5 minutes to pellet bacteria. The supernatant was removed and the pellet resuspended in PBS (phosphate-buffered saline) to reduce endotoxin transfer. 50 μL was transferred into 5 mL fresh culture medium (100-fold dilution) and at each transfer, 2 μL of bacteriophage stock was inoculated into relevant flasks. At each transfer, cultures were frozen (500 μL was frozen with 500 μL 50% glycerol at −70°C) and bacteriophage extracted via chloroform extraction as above. Bacteria and bacteriophage densities were estimated as previous. No-bacteriophage controls were monitored for bacteriophage presence. Three macrophage-only and two bacteria-only treatment replicates became contaminated during experimental evolution and were removed.

### Macrophage-bacteriophage effects on bacterial density

We characterized short-term interactions between bacteria, bacteriophage and macrophage to elucidate direct and indirect effects. *P. aeruginosa* was cultured overnight in 6 mL LB at 37°C. Macrophages were seeded into 6-well plates at a density of 7.5 × 10^5^ cells / well with 2.5 mL culture medium and incubated overnight to reach ~80%–90% confluency for experiments. For bacteria experiments, four treatments were established: bacteria only, bacteria with macrophages, bacteria with bacteriophages, and bacteria with macrophages and bacteriophages. *P. aeruginosa* was diluted to ~10^5^ CFU/ μL (0.1 OD) into PBS and 10 μL was inoculated into each microcosm (for macrophage cultures: ~1.5:1 macrophage to bacteria). To cultures containing bacteriophage, 10^5^ PFUs of each bacteriophage were inoculated (1 μL of each bacteriophage stock at 10^5^ PFUs/μL) to achieve an MOI of 0.1 per bacteriophage (bacteria to bacteriophage ratio). For bacteriophage experiments, two treatments were established: bacteriophage only, and macrophages with bacteriophages. 10^7^ PFUs (50 μL of 2 × 10^5^ stock bacteriophage) was inoculated (for macrophage cultures: 1:10 macrophage to bacteriophage ratio) into each well. Plates were incubated in tissue culture incubators for 8 hrs. At the end of the experiment, cultures were mixed via pipetting and 500 μL was frozen with 500 μL 50% glycerol at −70°C. To estimate changes in bacterial density, cultures were plated from frozen onto LB agar and incubated overnight at 37°C. Bacteriophage extracted from the bacteriophage experiment via chloroform extraction: 900 μL of culture was vortexed with 100 μL chloroform. Vials were then centrifuged at 14000 rpm (21 100× g; Progen GenFuge 24D centrifuge) for five minutes and the supernatant isolated. Bacteriophage density was estimated via spot assays: dilutions were spotted onto overlays containing 100 μL of *P. aeruginosa* (grown overnight as above) in 5 mL soft LB agar.

### Bacteriophage growth rate assay

We determined if macrophages affected the growth rates and relative competition of the two bacteriophages in our cocktail: 14–1 and PNM. To determine whether effects were driven by macrophage presence (direct effect) or by macrophages altering the media (indirect effect), we generated spent media from macrophage sub-culturing and activated macrophage media. Activated macrophage media was generated by exposing macrophages (2 × 30 mL 175 cm^3^ flasks of macrophages at 90% confluency) to heat-killed *P. aeruginosa* CN573. *P. aeruginosa* was first grown overnight in LB broth before being pelleted and washes 2× in PBS as before remove metabolites and media. The pellet was resuspended in PBS and then vials were placed at 80°C for 30 minutes (time selected based on preliminary experiments). 1.8 mL of heat-inactivated bacteria was then added to each flask. Macrophages were incubated for 24 hrs as previous. After 24 hrs, media was removed and first spotted onto LB agar to confirm no bacteria contamination, before being filter sterilized using 0.45 μm and 0.22 μm syringe filters to remove cellular debris. Media was stored at −20°C and thawed immediately prior to experimentation. For the macrophage-present treatment, macrophages were seeded into 6 25 cm^3^ flasks and grown overnight as previous to reach 90% confluency – media was refreshed prior to experimentation.

*P. aeruginosa* CN573 was cultured overnight, washed in PBS and diluted to 0.1 OD_600_ as previous. A master-mix of both bacteriophages was generated containing 1000 PFUs / μL of each bacteriophage. 20 μL of *P. aeruginosa* and 40 μL of bacteriophage master-mix was inoculated into independent flasks that contained either fresh DMEM, spent macrophage media, activated macrophage media, or macrophages. 5 independent replicates were set up for each treatment. Assays were run for 24 hrs. Samples were frozen and bacteriophage extracted via chloroform extraction as previous.

The density of each bacteriophage was determined by plating extractions onto lawns of three *P. aeruginosa* CN573 genotypes (wild-type (ancestor), PNM resistant, and 14–1 resistant). *P. aeruginosa* CN573 strains resistant to PNM and 14–1 were generated by inoculating 60 μL of *P. aeruginosa* into 6 mL LB with 10 μL stock of either 14–1 and PNM. Cultures were grown overnight before being streaked onto LB agar and grown overnight. Colonies were picked and resistance to each bacteriophage was confirmed by spot assays. Resistance to one bacteriophage did not affect plating efficiency of the other bacteriophage it was still susceptible to. The growth rate (m) of each bacteriophage was estimated as ln(N_1_/ N_0_) where N_1_ is the final density, N_0_ is starting density. The relative fitness of each bacteriophage was calculated by dividing the growth rate of 14–1 by the growth rate of PNM.

### Estimating coevolution

To estimate whether bacteria and bacteriophage have coevolved in isolation or in the presence of macrophages, we performed time-shift assays [17]. From Days two, four, and six, twelve colonies were isolated from each treatment replicate. To remove bacteriophage contamination, single colonies were resuspended in 10 μL M9 and were serially streaked onto LB plates for three transfers. Colonies were then grown overnight at 37°C and bacteriophage spot assays were performed to test resistance against bacteriophage isolates from Days two, four, and six. Bacteria isolates from Day 6 were tested for resistance against ancestral bacteriophages 14–1 and PNM. If bacteria and bacteriophage have coevolved via arms-race dynamics, we would expect bacteria to be more resistant to bacteriophages from the past and more susceptible to bacteriophages from the future. Twelve colonies were isolated from each no-bacteriophage treatment replicate (bacteria alone and bacteria with macrophages Day 6) and tested for bacteriophage resistance to 14–1 and PNM.

### Sequencing

Ancestral bacteriophages 14–1 and PNM were sequenced to identify any mutations which may have occurred which distinguish our samples from the reference genome. Prior to DNA extraction, cultures were treated with DNase to remove bacteria DNA. The NEB monarch genomic DNA extraction kit was used to extract gDNA and the concentration was determined as above. Sequencing was done by the University of Liverpool’s Centre for Genomic Research (CGR) using the PacBio Sequel IIe platform to generate an estimated average of 10 Gb HiFi data per library. Genomes were assembled and annotated using BV-BRC (Bacterial and Viral Bioinformatics Resource Center) bioinformatic tools (v 3.46.3) using Canu [36]. Genomes were annotated using the “bacteriophages” tool kit with comparison to the reference genomes of 14–1 (taxon ID: 581037) and PNM (taxon ID: 2975222).

The *P. aeruginosa* CN573 strain was sent for sequencing to MicrobesNG who extracted gDNA, sequenced and assembled the genome using in-house protocols. Sequencing libraries were generated using SQK-RBK114.96. Sequencing was performed on a GridION (Oxford Nanopore Technologies) using an R10.4.1 flowcell, with basecalling model r1041_e82_400bps_sup_variant_v4.3.0. Reads were randomly subsampled to 50× coverage using Rasusa (V 0.7.1), and assembled using Flye (V2.9.2-b1786). Assemblies were polished using Medaka (V 1.11.3) and the relevant model (r1041_e82_400bps_sup_variant_v4.3.0). Genomes were annotated using BV-BRC annotation tools with comparison to the reference genome *P. aeruginosa* PAO1 (identified as the closest characterized *P. aeruginosa* genome).

Bacteria populations from each treatment replicate from Day 6 were analysed for mutations. The 12 isolates isolated from each treatment replicate were pooled by mixing one colony of each isolate into extraction buffer. Pooled isolates were used to remove bacteriophage contamination, which would have been present in original stocks taken from experimental evolution. Bacteriophage populations from Days 2 and 6 were amplified from original stocks by inoculating 150 μL stock into 15 mL LB with 150 μL overnight culture of *P. aeruginosa* CN573. Cultures were grown overnight, shaking, at 180 r.p.m. Bacteriophage were extracted by filtering cultures through 0.22 μm syringe filters. Bacteriophages were amplified and treated with DNase as above. The Qiagen DNeasy Ultra Clean DNA Extraction Kit reagents and protocol were used to extract the gDNA from the cultures. The concentration of gDNA in each sample was determined using the QuBit dsDNA HS Assay kit regents and protocol. Sequencing was done by the University of Liverpool’s CGR using an Illumina Novaseq to create 150 bp paired end reads. The CGR trimmed for the presence of Illumina adapter sequences using Cutadapt (v1.2.1) and removed reads with a minimum quality score of 20 and which were shorter 15 bp in length. Variant analysis on Day 6 populations was run using BV-BRC tools with bacteria and bacteriophages compared to the assembled genomes of the ancestral strains. BWA-mem was used to align reads and FreeBayes was used to identify high-quality variants. Prior to analysis, variants found in every read library were identified as sequencing errors and removed.

### Growth rate assay

To determine any costs of resistance we measured exponential growth rates. The same bacteria isolates that were used in the biofilm assay were used. Overnight cultures were diluted to 0.1 OD_600_ into PBS. 10 μL of diluted cultured was inoculated into 190 μL LB media in 96-well plates. Although bacteria had evolved in a different media type (DMEM), LB was used since DMEM contains phenol red that would have likely interfered with optical density estimates. The plate was incubated in a spectrophotometer (MultiSkan Sky) at 37°C in shaken conditions and OD_600_ was measured every 20 min for 24 hrs to estimate changes in cellular density. Growth curves were independently replicated three times (technical replicates). Reads after 5 hrs of growth were removed as populations reached stationary phase with fluctuations in density estimates (likely due to biofilm formation).

### Biofilm assay

As strain CN573 is mucoid, we considered whether bacteriophage resistance or evolution with macrophages had altered biofilm production – reductions in biofilm can make bacteria more vulnerable to the immune system. Six bacteriophage resistant and six bacteriophage susceptible isolates was selected from independent treatment replicates from macrophage present and macrophage absent treatments (three resistant/susceptible from each treatment). These were compared to three replicates of the ancestral bacteria, and three isolates from the bacteria-only evolution lines, and the bacteria-macrophage (no bacteriophage) evolution lines, all isolated from independent replicates. Biofilm production was estimated using a resazurin assay, based upon a previously described protocol [37]. Overnight monocultures were diluted to 0.1 OD_600_ (~10^9^ CFU/mL) into PBS. 100 μL of each culture was inoculated into a sterile 96-well plate and incubated at 37°C for 4 hrs for cellular adhesion. Then, the supernatant was removed, and wells were washed gently with 100 μL of PBS by pipetting up and down three times. Next, 100 μL of fresh LB media was added to each well, including the controls (no bacteria) and plates were incubated for 16 hrs. The liquid was then removed and wells were washed with 100 μL PBS. 100 μL of fresh M9 was added followed by 20 μL Cell Titre Blue. Fluorescence (λex: 560 nm and λem: 590 nm) was measured after 2-hr incubation. Biofilm assays were independently replicated three times (technical replicates).

### Inflammatory cytokine production

We next assessed whether bacteriophage resistance led to changes in inflammatory cytokine production – specifically TNF-α as a key mediator of inflammation, which is produced in response to bacterial infection. Six-well plates were seeded with 7.5 × 10^5^ macrophages and grown for 24 hrs to reach 90%–100% confluency and media was refreshed. Bacteria were grown overnight (same isolates as used in the biofilm and growth rate assay) and diluted to 0.1 OD_600_ into PBS and 15 μL was inoculated into independent cultures (macrophage to bacteria ratio approx. 1:1). Three macrophage cultures were not inoculated and kept as controls. Cultures were grown overnight in a tissue culture incubator as above. After culturing, 1 mL of culture was filtered through 0.45 μm and 0.22 μm syringe filters to remove bacteria and cellular debris. Supernatants were stored at −80°C and were thawed immediately prior to cytokine quantification. TNF-α concentrations were quantified using a ThermoFisher ELISA kit for mouse cytokines following manufacturer instructions. Samples were pre-diluted 100× so that values fit into the standard curve (identified in preliminary analysis). Samples were analysed in duplicate including blank controls—all results fit within 20% of the mean value. Absorbance OD_450_ and background absorbance OD_620_ was measured using spectrophotometer (MultiSkan Sky). A non-linear model was fitted to the standard curve of known TNF-α standard concentrations and resultant OD (after subtracting the background absorbance). TNF-α concentrations in the samples were estimated by predicting values based on the standard curve and multiplying by the dilution factor.

### Statistical analyses

All data were analysed using R (v.4.2.1) in RStudio [38], all plots were made using the package “*ggplot2*” [39] and all tables were made using the package “*flextable*” [40]. Model simplification was conducted using likelihood ratio tests and Tukey’s post hoc multiple comparison tests were used to identify the most parsimonious model using the R package “*emmeans*” with *P*-values adjusted using the Tukey method [41]. In experimental evolution, bacterial density (log10(CFU/mL)) was tested against interacting fixed effects of macrophage presence, bacteriophage presence and time, with a random effect of treatment replicate. Changes in bacteriophage density was analysed in a linear mixed effects model analysing density (log10(PFU/mL)) against interacting fixed effects of time and macrophage presence with a random effect of treatment replicate.

Bacterial density (after 8 hrs culturing) [log10(CFU/mL)] in short-term assays was analysed against interacting fixed effects of macrophage and bacteriophage presence in a linear model. Bacteriophage density after culturing with macrophages alone was determined in a linear model analysing bacteriophage density [log10(PFU/mL)] against macrophage presence. Growth assays of bacteriophages 14–1 and PNM were analysed in a linear model with relative fitness was analysed against treatment (media type / macrophage presence). Additionally, the bacteriophage growth rates (*m*) were analysed against interacting fixed effects of treatment and bacteriophage identity (14–1 or PNM) with a random effect of treatment replicate.

Bacteriophage resistance to ancestral bacteriophage 14–1 on Day 6 was analysed in a generalized linear model with a fixed effect of macrophage presence with a quasibinomial error structure. To assess whether bacteria and bacteriophage coevolved in the presence or absence of macrophages, a binomial generalized mixed effects linear model was used. Here, the proportion of bacteriophage resistant bacteria was analysed against interacting fixed effects of bacterial time and bacteriophage time with a random effect of treatment replicate. Separate models were constructed for each treatment (macrophages present or absent) to improve model fitting and primarily characterize whether coevolution occurred independent of treatment comparisons.

Bacteriophage populations were analysed based on the genetic distance from the ancestral population, calculated as the sum of the difference of the proportion of each SNP/indel in each population from the ancestral proportion. Genetic distance was analysed in a linear model with a fixed effect of macrophage presence. Changes in frequency of PNM in the bacteriophage cocktail was measured by analysing the proportion of reads that mapped to PNM (arcsine transformed) against a fixed effect of day (two or six) in a linear mixed effects model with a random effect of treatment replicate. The relationship between the proportion of bacteriophage resistant bacteria (taken as the average resistance to bacteriophage isolated from Days 2 and 6 of the experiment) and proportion of PNM was analysed in a generalized linear model with a binomial error structure and a random effect of treatment replicate.

Biofilm production was log transformed [log(fluorescence λex: 560 nm and λem: 590 nm)] and analysed against interacting fixed effects of treatment (which evolution treatment clones originated from e.g. bacteria only, bacteria with macrophages etc.) and bacteriophage resistance. Growth rates were estimated using a rolling regression taking the steepest slope of the linear regression between ln OD_600_ and time in hours in a shifting window of every four time points (every 1.3 hrs). Growth rate was averaged over each technical replicate for each biological replicate (individual bacterial isolates). Differences in growth rates were analysed against interacting fixed effects of treatment and bacteriophage resistance in a linear model. Additionally, biofilm production was analysed against growth rates in a linear model.

We analysed whether there were any significant differences in TNF-α cytokine production by macrophages when exposed to different bacteria populations. First, we analysed whether cytokine production (log transformed) differed across treatment groups including the macrophage control (unstimulated macrophages) in a linear model. Next, we assessed whether there were any differences in cytokine production between bacteriophage resistant and bacteriophage susceptible isolates originating from macrophage and bacteriophage treatment and bacteriophage only treatment. In a linear model, cytokine production was analysed against interacting fixed effects of macrophage and bacteriophage presence (during evolution).

## Results

### Bacteria and bacteriophage densities during experimental evolution

**Figure 2:**
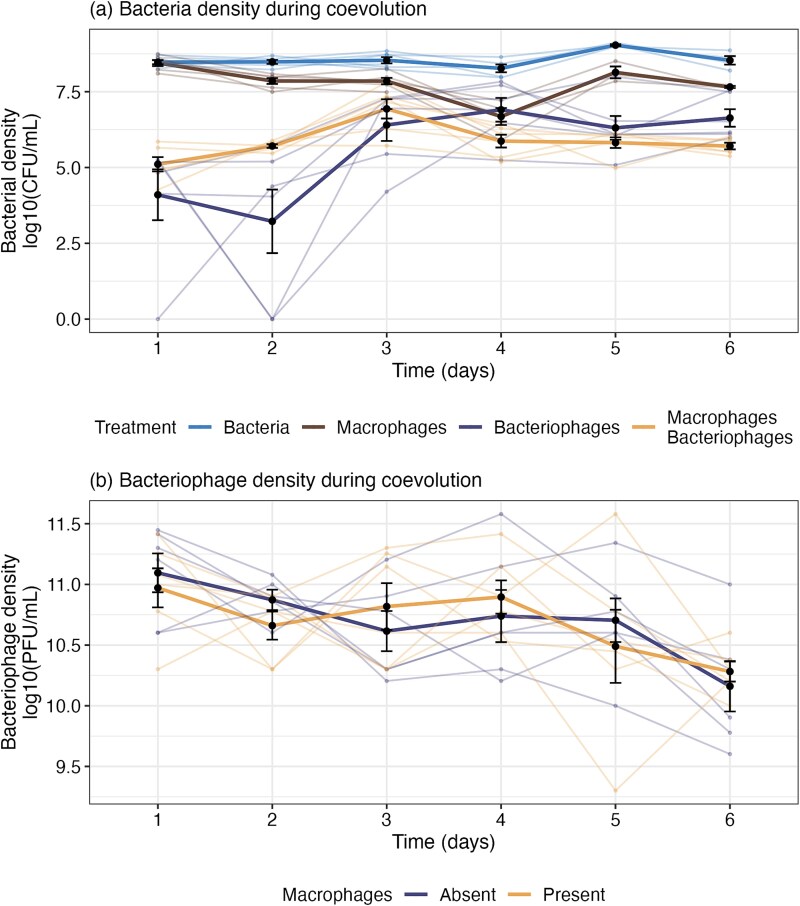
Ecological and evolutionary dynamics observed over experimental evolution. (a) Bacterial density [log10(CFU/mL)] in the presence and absence of bacteriophages and macrophages; and (b) bacteriophage density [log10(PFU/mL)] in the presence and absence of macrophages. Lines connect individual treatment replicates and changes in the treatment mean over time. Small points indicate individual treatment replicates. Large points show the treatment mean. Bars indicate standard error (± SE).

Reducing, with the aim to clear, bacterial populations is the key factor in phage therapy success; therefore, in vitro population dynamics of bacteria and bacteriophage provide a simplified model that can help explore how immune components impact phage therapy efficacy. In the 1–2 days of treatment, bacteriophages primarily decreased bacterial densities (10^3.67^ CFUs/mL, ±0.94 SE) relative to controls (10^8.48^ CFUs/mL, ±0.07 SE; ANOVA comparing models with and without the three-way interaction between time, macrophage and bacteriophage presence: ${x}_5^2$ = 13.34, *P* = .0204; Table S2; Fig. 2a). Macrophages alone had no significant effect on bacterial densities (Tukey HSD: *P* > .05; Table S2). By Day 2, macrophage and bacteriophage interacted antagonistically with bacterial densities in the presence of macrophages and bacteriophages being ~300-fold higher ($\overline{x}$ = 5.71, 95%CI = 4.99–6.43) than in the bacteriophage-only treatment ($\overline{x}$ = 3.23, 95%CI = 2.51–3.94; Tukey HSD: t-ratio = −4.84, *P* < .001).

However, from Day 3 onwards, bacterial populations with bacteriophage-only and bacteriophages and macrophages increased significantly. Here, densities increased by 1504-fold with bacteriophages, and increased 16.8-fold with bacteriophages and macrophages. While densities with bacteriophages or “macrophages and bacteriophages” were significantly lower than controls (bacteria only and macrophages), there were no significant differences between bacteriophage treatments from Day 3 onwards (Table S2, Fig. 2a).

Bacteriophage density was not significantly affected by macrophages (ANOVA comparing models with and without macrophage x time: ${x}_5^2$ = 3.68, *P* = .596; fixed effect of macrophage presence: ${x}_1^2$ = 0.01, *P* = .939) and were stable across most time-points (Tukey HSD comparisons between time-points 1–5: *P* > .05; Table S3; Fig. 2b). However, by Day 6, bacteriophage densities showed significant decline in both treatments (Tukey HSD comparisons between time-points 1–5 and 6: *P* < .05; Table S3; Fig. 2b).

### Macrophages and bacteriophages interact antagonistically over short timescales

**Figure 3:**
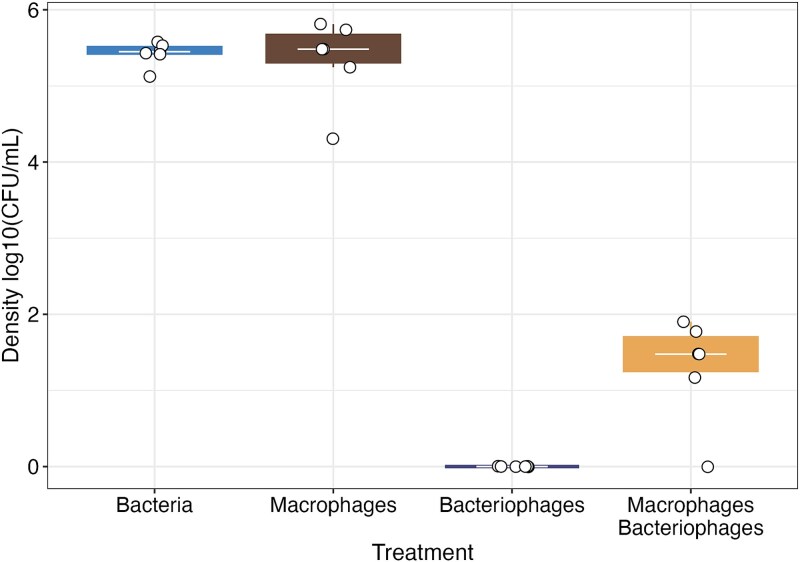
The effects of macrophages and bacteriophages on bacterial density over 8 hrs of bacterial growth. Tops and bottoms of the bars represent the 75th and 25th percentiles of the data, the middle lines are the medians, and the whiskers extend from their respective hinge to the smallest or largest value no further than 1.5^*^ interquartile range.

To understand the mechanisms influencing the changes in bacterial densities and (co)evolution, we measured the effect of bacteriophages and macrophages over even shorter time scales (8 hours) (preceding any resistance evolution and significant macrophage death). Bacterial densities with macrophages and bacteriophages were significantly higher ($\overline{x}$ = 1.3, 95%CI = 0.921–1.683) than when bacteria were cultured with bacteriophage alone (cultures below detectable density; ANOVA comparing models with and without macrophage x bacteriophage presence interaction: ${x}_1^2$ = 14.32, *P* = .001; Tukey HSD; Fig. 3). Comparisons between the bacteria-only control and bacteriophage alone (Tukey HSD: t-ratio = 21.01, *P* < .001) and macrophage-bacteriophage treatment (Tukey HSD: t-ratio = 15.97, *P* < .001) were significant (Fig. 3). Macrophages alone did not significantly decrease bacterial densities compared to controls (Tukey HSD: t-ratio = 0.31, *P* = .989; Fig. 3).

This result again indicated antagonism between bacteriophages and macrophages in how they affect bacteria density. We considered whether macrophages directly degraded bacteriophages by culturing bacteriophages alone with macrophages (no bacteria present). Macrophages were not found to significantly affect bacteriophage densities over 8 hrs of culturing (*F*_1,9_ = 0.04, *P* = .839; Fig. S3). This result is also consistent with previous work, which found that macrophages did not affect bacteriophage densities based on plate counts but did interfere with bacteriophage infection by phagocytosing bacteriophages [14].

**Figure 4:**
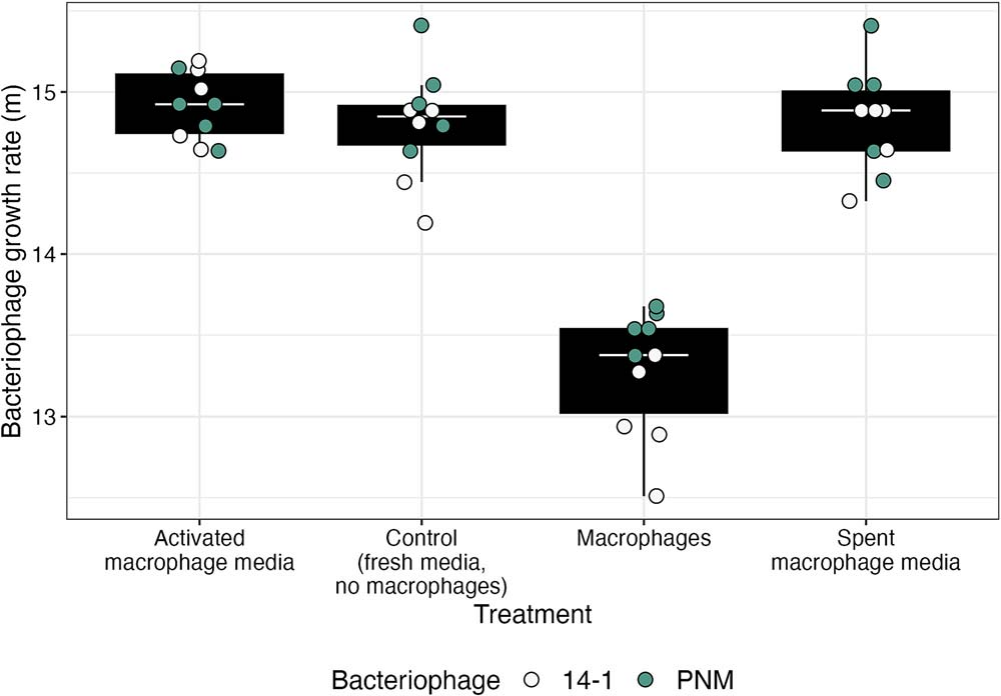
Bacteriophage (phage) growth rates (m) in different media and macrophage treatments. Tops and bottoms of the bars represent the 75th and 25th percentiles of the data, the middle lines are the medians, and the whiskers extend from their respective hinge to the smallest or largest value no further than 1.5^*^ interquartile range.

Instead, macrophages were found to directly inhibit the growth rates of both bacteriophages. We grew 14–1 and PNM in normal media (Dulbecco’s Modified Eagle’s Medium), spent macrophage media (from non-activated macrophages), media from activated macrophages (stimulated with heat-killed *P. aeruginosa*), and in fresh media with macrophages present (as set up in the original evolution experiment). Neither macrophages nor media type significantly affected the relative fitness between the bacteriophages when in competition (*F*_3,16_ = 1.95, *P* = .162, Fig. S6). However, the presence of macrophages did significantly decrease the growth rates of the bacteriophages (ANOVA comparing models with and without treatment: ${x}_3^2$= 57.97, *P* < .001; Tukey HSD comparisons: *P* < .001, Table S4, Fig. 4) suggesting that macrophages can inhibit bacteria-phage contact rates to some extent. This effect is greater, but consistent with the non-significant effect of macrophages on bacteriophage densities observed in the first two days of the coevolution experiment. The growth rate of 14–1 ($\overline{x}$ = 14.3 *m*, 95% CI = 14.2–14.5) was on average lower than PNM ($\overline{x}$ = 14.6 *m*, 95% CI = 14.4–14.7; ANOVA comparing models with and without bacteriophage identity: ${x}_3^2$ = 7.71, *P* = .005; Tukey HSD comparing growth rates of 14–1 to PNM: estimate = −0.25, t-ratio = −2.73, *P* = .013), however this difference was not significantly affected by treatment (ANOVA comparing models with and without bacteriophage identity and treatment interaction: ${x}_3^2$ = 7.3, *P* = .063; Fig. 4).

### Bacteria-phage coevolution

**Figure 5:**
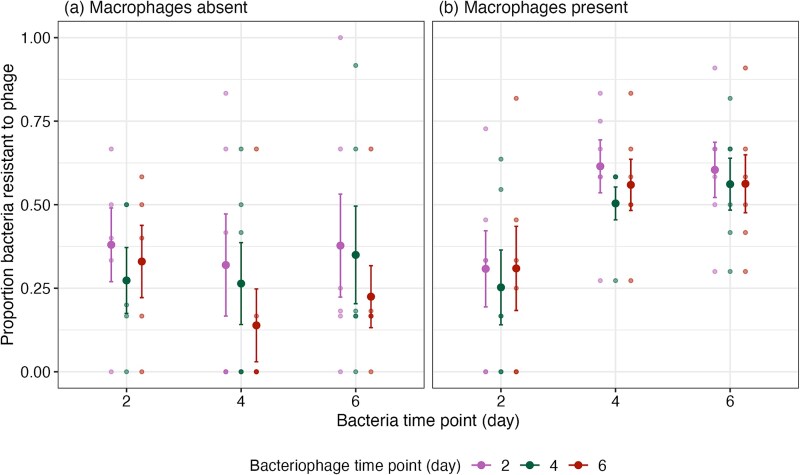
Time-series assay analysing the proportion of bacteria resistant to bacteriophages from different time-points where macrophages are (a) absent and (b) present. Small points indicate individual treatment replicates and large points show the treatment mean. Bars indicate standard error (± SE).

We next determined how the patterns of bacteria-phage (co)evolution were affected by macrophages, and if these patterns could explain the convergence of ecological dynamics through time. Bacteria-bacteriophage (co)evolution was measured using a time-shift assay in which bacteria from different time-points are exposed to bacteriophages from different time-points (Fig. 1). In the absence of macrophages, 32.8% (5.8 ± SE) of bacteria isolates were bacteriophage resistant by Day 2 and this did not significantly increase through time (Day 4 = 24.1%, 7.3 ± SE; Day 6 = 31.7%, 7.4 ± SE; ANOVA comparing models with and without bacteriophage x bacteria time interaction: ${x}_4^2$ = 4.44, *P* = .350; ANOVA comparing models with and without bacterial time-point: ${x}_2^2$ = 4.64, *P* = .098; Fig. 5a). The bacteriophage cocktail significantly increased in infectivity through time (ANOVA comparing models with and without bacteriophage time: ${x}_2^2$ = 9.76, *P* = .008; Tukey HSD of bacteriophage infectivity between Days 2 and 6: z-ratio = 3.073, *P* = .006). This pattern is broadly indicative of asymmetrical coevolution with increases in bacteriophage infectivity occurring without increases in bacterial resistance.

Where macrophages were present, the proportion of bacteriophage resistant bacteria increased through time from 29% (6.4 ± SE) resistant at Day 2 to 57.6% (8.2 ± SE) resistant at Day 6 (ANOVA comparing models with and without bacterial time-point: ${x}_2^2$ = 48.51, *P* < .001; Tukey HSD: estimate = −1.301, z-ratio = −6.05, *P* < .001). Unlike the no-macrophage treatment, the bacteriophage cocktail did not increase in infectivity through time (ANOVA comparing models with and without bacteriophage x bacteria time interaction: ${x}_4^2$ = 0.662, *P* = .956; ANOVA comparing models with and without bacteriophage time: ${x}_2^2$ = 2.45, *P* = .294; Fig. 5b). Therefore, the presence of macrophages limited coevolution with only increases in the overall rates of bacteriophage resistance without changes to bacteriophage infectivity.

These results demonstrate macrophages affected patterns of bacteriophage resistance and changes in bacteriophage cocktail efficacy. These different patterns led to differences in contemporary resistance i.e. the resistance of bacteria to the population of bacteriophages isolated from the same time point (e.g. resistance of bacteria from Day 6 to bacteriophages from Day 6). Where macrophages were absent, contemporary bacteriophage resistance was significantly lower (Day 4: $\overline{x}$ = 0.27, 95%CI = 0.164–0.420; Day 6: $\overline{x}$ = 0.22, 95%CI = 0.125–0.353) compared to where macrophages were present at Days 4 (macrophages absent: $\overline{x}$ = 0.27, 95%CI = 0.164–0.420; macrophages present: $\overline{x}$ = 0.51, 95%CI = 0.363–0.650, Tukey HSD: estimate = 1.004, z-ratio = 2.24, *P* = .025) and six (macrophages absent: $\overline{x}$ = 0.22, 95%CI = 0.125–0.353; macrophages present: $\overline{x}$ = 0.57, 95%CI = 0.422–0.708, Tukey HSD: estimate = 1.56, z-ratio = 3.40, *P* < .001; ANOVA comparing models with and without treatment x time interaction: ${x}_2^2$ = 13.48, *P* = .001; Fig. 5). This effect was not present at Day 2, however this was before bacteria in macrophage-present cultures increased in bacteriophage resistance, and before the bacteriophage cocktail increased in infectivity in the macrophage-absent cultures (Tukey HSD: estimate = −0.398, z-ratio = −0.85, *P* = .395). No bacteriophage resistance emerged against the ancestral bacteriophage PNM in all macrophage replicates (Fig. S4). In the absence of macrophages, PNM resistance occurred in 2/6 treatment replicates at Day 6 (16.7% and 8.3%). Bacteriophage resistance at Day 6 to ancestral 14–1 was non-significantly different between macrophage-present cultures (56.3%, 7.6 ± SE) compared to when bacteria were cultured with bacteriophages alone (30.6%, 14.5 ± SE; *F*_1,10_ = 1.5, *P* = .247; Fig. S4). In summary, resistance evolved in both the presence and absence of macrophages, but with macrophages promoting greater evolved resistance to bacteriophage and hindering increases in bacteriophage cocktail efficacy.

### Genetic changes within bacteria and bacteriophage populations

Sequencing analyses were conducted to determine the genetic underpinnings of bacteria-phage (co)evolution (Fig. 1). Few mutations were found in bacteria populations (Fig. S5). No mutations were found in bacteria-only and bacteria-macrophage (no bacteriophage) treatments. In treatments containing bacteriophages, mutations were found in three out of six replicates of each treatment (six total, macrophage present and absent). In four out of six of these populations, one missense or frameshift mutation was found in a hypothetical protein upstream of a gene associated with LPS biosynthesis (Fig. S5;  Supplementary  File  S1). As 14–1 infects via LPS therefore, it is likely this mutation is associated with 14–1 resistance. Only one population had more than one mutation (three) including in genes associated with pili maturation and two hypothetical proteins (one in a region of chloramphenicol resistance genes and the other upstream of LPS biosynthesis)—this population was the only treatment replicate in which resistance to 14–1 reached fixation suggesting multiple mutations were required for this to occur (Fig. S5). The remaining bacterial populations had mutations at a hypothetical protein associated with a chloramphenicol resistance region (upstream and downstream genes) and dihydrootase, which are responsible for pyrimidines nucleotide synthesis—the mechanistic link between these mutations and bacteriophage resistance is unclear (Fig. S5). Additionally, some populations, which had bacteriophage resistant isolates, had no genetic mutations. This suggests that resistance also occurred via mechanisms such as phase variation in which the expression of genes, such as for bacteriophage receptors, is turned off [42, 43]. The presence of restriction modification systems in this strain may have further contributed to phase variation, and/or phage defence [44]. These alterations are heritable but are not identifiable via nucleotide sequencing [42, 43].

**Figure 6:**
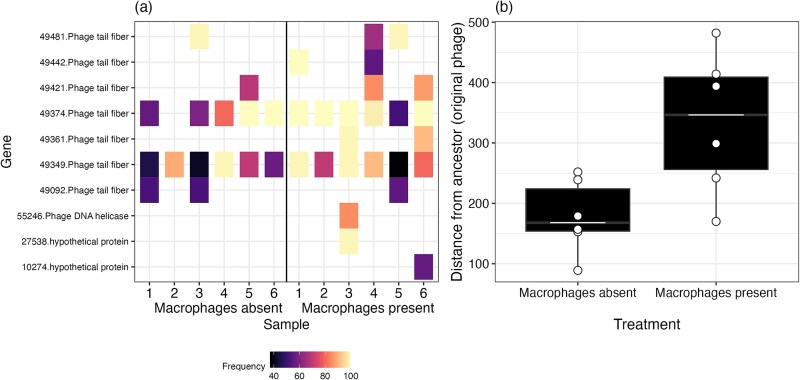
Bacteriophage (14–1) evolution in the presence and absent of macrophages at Day 6. (a) In the presence of macrophages, a greater number of unique genetic variants are selected and increase to higher frequencies (%) within the population. On the y-axis the number indicates the position of the genetic variant within the genome while the gene name indicates the type of gene the mutation occurred in. 1–6 on the x-axis indicates the replicate number of each treatment. (b) these evolutionary differences can be summarized into the overall distance from the ancestor (sum of genetic variants and their frequencies) which shows that bacteriophages diverge more from the ancestor when macrophages are present. Points indicate individual treatment replicates. Tops and bottoms of the bars represent the 75th and 25th percentiles of the data, the middle lines are the medians, and the whiskers extend from their respective hinge to the smallest or largest value no further than 1.5^*^ interquartile range.

Next, we considered the bacteriophage populations. Overall, our sequencing analysis of PNM populations revealed no mutations compared to the ancestor while 15 and 24 mutations were found 14–1 populations evolved in the absence and presence of macrophages respectively. Most mutations (36/39) were missense mutations in bacteriophage tail fibre proteins while the remaining mutations (3/39) were in DNA helicase (missense mutation) and two hypothetical proteins (one missense, one conservative in-frame deletion; Supplementary File S2). Time-shift assays of coevolution suggested that bacteriophages increased in infectivity where macrophages were absent. If this effect was driven by genetic adaptation in the bacteriophage, we would expect more mutations in the macrophage-absent populations. Contrary to our predictions, 14–1 evolved significantly more in the presence of macrophages ($\overline{x}$ = 334, 95% = 248.8–418) compared to when macrophages were absent ($\overline{x}$ = 178, 95% CI = 93.5–263; ANOVA comparing models with and without macrophage presence: ${x}_1^2$ = 8.35, *P* = .016; Tukey HSD comparing genetic distance between bacteriophage populations from macrophage absent to present cultures: estimate = −155, t-ratio = −2.89, *P* = .016; Fig. 6).

Differences in bacteriophage resistance and patterns of coevolution may be in-part explained by differences in PNM frequencies between macrophage present and absent cultures, in addition to any evolutionary change. Sequencing showed that PNM was present in only 1/6 macrophage-present replicates, while 4/6 treatment replicates contained PNM when macrophages were absent. The absence of PNM in macrophage-present cultures explains why there was no resistance evolution to this bacteriophage in these replicates, while resistance to PNM emerged where macrophages were absent in 2/6 treatment replicates. Additionally, where macrophages were absent, the percentage of reads mapping to PNM increased from 28.3% (6.65 ± SE) at Day 2 to 35.01% (12.2 ± SE) at Day 6. Although the increase was statistically non-significant (ANOVA comparing models with and without time: ${x}_1^2$ = 0.718, *P* = .397), 4/6 treatment replicates showed an increase in PNM proportion between Days 2 and 6. Therefore, considering that most bacteria remained susceptible to PNM, it is likely that changes in PNM frequency also contributed to increases in bacteriophage cocktail infectivity. Since there was no statistical relationship between the frequency of bacteriophage resistant bacteria and proportion of PNM at each time point (ANOVA comparing models with and without PNM proportion: ${x}_1^2$ = 1.01, *P* = .316), there are likely to be other variables influencing bacteriophage cocktail infectivity through time.

### Phenotypic effects of bacteriophage resistance

**Figure 7:**
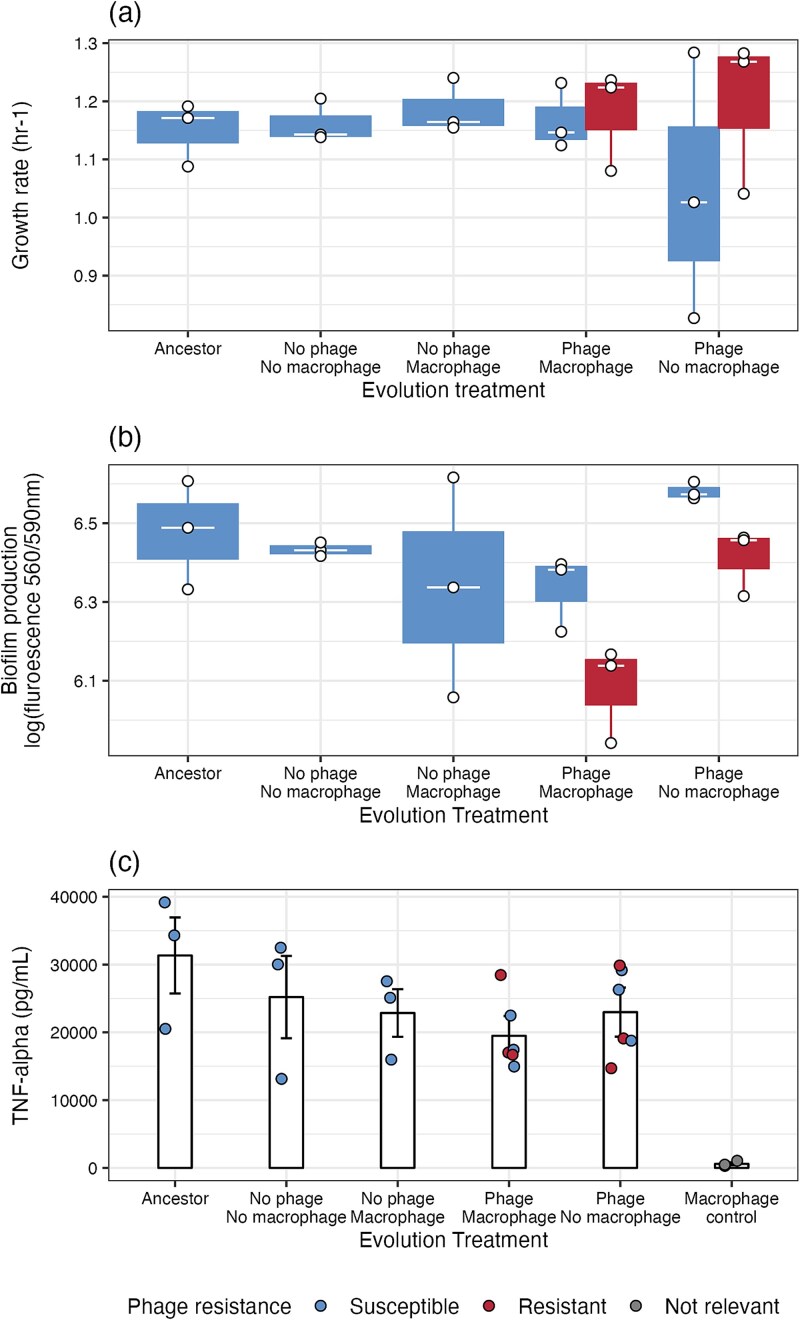
Phenotypic changes in bacteria associated with bacteriophage (phage) resistance and treatment bacteria evolved in during experimental evolution including (a) growth rates, (b) biofilm production and (c) macrophage stimulation, measured in production of pro-inflammatory cytokine production (TNF-α); bars indicate the treatment mean while error bars represent ± SE. Points indicate individual treatment replicates. Tops and bottoms of the bars represent the 75th and 25th percentiles of the data, the middle lines are the medians, and the whiskers extend from their respective hinge to the smallest or largest value no further than 1.5^*^ interquartile range.

Finally, we considered the phenotypic consequences of bacteriophage resistance as these can directly affect bacteria and bacteriophage population dynamics and how bacteria might affect an infected host. We were unable to detect a cost of resistance, regardless of whether resistant isolates originated from bacteriophage-only or bacteriophage-macrophage treatments (ANOVA comparing models with and without resistance x treatment interaction: *F*_1, 14_ = 1.2, *P* = .302; resistance: *F*_1, 19_ = 0.8, *P* = .377; evolution treatments: *F*_4, 15_ = 0.5, *P* = .769; Fig. 7a).

We also considered biofilm formation, which can affect virulence, antibiotic resistance and macrophage stimulation (Fig. 1). On average, bacteriophage resistant populations produced significantly less biofilm ($\overline{x}$ = 6.22 log(fluorescence), 95%CI = 6.08–6.37) than bacteriophage susceptible populations ($\overline{x}$ = 6.43, 95%CI = 6.36–6.51; ANOVA comparing models without and without bacteriophage resistance: *F*_1, 15_ = 7.6, *P* = .015; Tukey HSD: estimate = 0.21, t-ratio = 2.75, *P* = .015; Fig. 7b). Furthermore, bacteria evolved with macrophages and bacteriophages produced less biofilm ($\overline{x}$ = 6.21, 95%CI = 6.09–6.32) than bacteria evolved with bacteriophages but no macrophages ($\overline{x}$ = 6.5, 95%CI = 6.38–6.61; ANOVA comparing models with and without treatment: *F*_4, 15_ = 4.1, *P* = .02; Tukey HSD: estimate = −0.289, t-ratio = −3.77, *P* = .014; Fig. 7b); suggesting some population phenotypic divergence under bacteriophage selection. All other comparisons between treatment groups were non-significant (Table S5). There was no correlation between growth rate and biofilm production (*F*_1, 19_ = 2.2, *P* = .152).

Next, we considered interactions between bacteria isolates and macrophages by measuring levels of pro-inflammatory cytokines (TNF-α). TNF-α did not differ between bacteriophage resistant or susceptible isolates (ANOVA comparing models with and without bacteriophage resistance: *F*_1, 9_ = 0.1, *P* = .829) or between isolates evolved with bacteriophages and with or without macrophages (ANOVA comparing models without and without evolution with macrophages: *F*_1,10_ = 1.1, *P* = .327; and the interaction: *F*_1,8_ = 0.8, *P* = .399; Fig. 7c). While there was a significant effect of treatment on cytokine production (ANOVA comparing models with and without evolution treatment: *F*_5,18_ = 57.2, *P* < .001), significant comparisons were only found between bacteria-present cultures and the macrophage-only control (no bacteria present) (Table S6). We also wanted to consider rates of phagocytosis, however due to the bacteria’s mucoid state it quickly forms biofilms resistant to antibiotic mediated killing (unpublished pilot work), making it difficult to eliminate extracellular bacteria.

## Discussion

Phage therapy outcomes are likely influenced by whether interactions between bacteriophage and the immune system are synergistic or antagonistic [11, 13, 18, 45], however how these interactions change from short to long term dynamics is poorly understood. Here, our results support a previous study showing that over short timescales (8 hrs and 1–2 days of experimental evolution), macrophages reduce the rate by which bacteria were killed by bacteriophages. Our growth rate assays with bacteriophages show that bacteriophage growth rates are inhibited when macrophages are present. Since this effect is dependent on the macrophages being physically present (no effect was observed using spent media) while not directly degrading bacteriophages, we hypothesize that the mechanism in part involved macrophages internalizing bacteriophages, thereby reducing infection rates (as in [14]). In mice, this antagonism resulted in greater exacerbation of infection symptoms, with mice reaching euthanasia end-points faster than mice without macrophages [14]. The presence of gene *Opr*F in this strain also presents the possibility that *P. aeruginosa* was evading bacteriophage infection by surviving within the macrophages [46]. However, over longer timescales, the antagonism between macrophages and bacteriophages diminished with bacteria and bacteriophage densities showing similar dynamics regardless of the presence of macrophages. This convergence is presumably driven by the rapid increase in bacteria densities (10–1500-fold) associated with the evolution of resistance to bacteriophages in both treatments obscuring macrophage effects. This study highlights that the effect of macrophages on bacteria-phage interactions can change over clinically relevant time scales.

While bacteriophage resistance rapidly evolved, when bacteriophage were present, contemporary levels of bacteriophage resistance were significantly higher when macrophages were also present. Differences in contemporary resistance were, in part, driven by differential patterns of coevolution. In the absence of macrophages, we observed an increase in bacteriophage cocktail infectivity when macrophages were absent between Days 2 to 6, which was not present when macrophages were present. This supports observations that macrophages gave bacteria a relative advantage in population density, which likely significantly increased bacterial mutation (including epigenetic potential) supply rates [15]. As bacterial densities were very low, at almost undetectable densities, when just bacteriophages were present with bacteria, mutation supply rates for bacteriophage resistance were lower. We note that different evolutionary outcomes observed within populations cannot explain why antagonism converged: resistance without significant costs was greater in the presence of macrophages that if anything should have led to increased, not decreased, antagonism between bacteriophages and macrophages.

Mechanisms of bacteriophage resistance did not appear to differ between treatments. Four out of twelve treatment replicates (two each for macrophage present/absent treatments) had mutations upstream of LPS which is likely associated with resistance to 14–1 (LPS binding). One macrophage-absent treatment replicate also had mutations in an intergenic region upstream of PilC—while PNM is pilus-binding, no resistance to PNM was found in this replicate, suggesting this may be a more general resistance mutation to 14–1 and potentially also associated with decreases in biofilm production observed. Few mutations were found, with many replicates that had bacteriophage resistant isolates having no detectable mutations—this raises the possibility that bacteriophage resistance also emerged by phase variation [43] which was undetectable in our sequencing analysis.

Bacteriophage population dynamics were also similar between macrophage present and absent cultures, with bacteriophage densities beginning to decline by Day 6 in both treatments. This result is not unexpected with the emergence of phage resistance, which limits susceptible host populations. However, these results are observed despite significant evolution of bacteriophage 14–1 (the dominant bacteriophage in all cultures). No mutations were found in PNM while significantly more 14–1 mutations were found in macrophage present cultures. These increased mutation rates may be driven by the increased rates of resistance, or in adaptation to macrophages. As most of the mutations were in tail fibres, this would suggest adaptation to resistant isolates as overcoming resistance is typically associated with changes to tail fibres that bind to mutated host receptors [47]. The propensity of the bacteriophages to adapt with immune cells present does open possibilities in future work to adapt phages to these *in vivo* conditions. That populations declined towards the end of the experiment further suggested that these mutations offered little adaptive benefit however in their contemporary populations.

While differences in resistance evolution by macrophages did not significantly alter bacteria population dynamics, there were different phenotypic effects of resistance on biofilm formation. Populations evolved with both macrophages and bacteriophages produced significantly less biofilm than those evolved with bacteriophages alone; a trait that can correlate with alterations in bacterial pathogenicity and chronic state. This was compounded by phage resistance, which led to further reductions in biofilm production, independent of macrophage presence or any differences in growth rate. However, we did not see evidence of decreased proinflammatory cytokine production in macrophages exposed to bacteriophage resistant (lower biofilm producing) populations. This suggests that phenotypic differences in bacterial populations did not lead to significant alterations in macrophage stimulation, or levels of toxicity towards macrophages that affected rates of cell death.

Our approach integrated immune components (in this case, a macrophage cell line) into commonly used experimental evolution systems that allow examination of bacteria and phage populations through time. An obvious caveat for wider interpretation is that conditions in vitro are more favorable for bacterial growth than *in vivo*, which may limit the ability of immune components to act as they quickly become overwhelmed with bacteria. Although were partially successful in mitigating this by refreshing macrophages at each transfer, macrophages *in vivo* may have greater antimicrobial potential through time. As such, we emphasize the general pattern that interactions among bacteria, phages and immune components are likely to change over evolutionary time, but not over these exact timescales.

Overall, our results support recent research that macrophages and bacteriophages behave antagonistically over the short term, but that that this antagonism decreases following the evolution of bacterial resistance to bacteriophages. Although macrophages increased the rate of phage resistance, this effect did not culminate in increased bacteria densities. In an *in vivo* setting, resistant populations might be removed by neutrophils [13], therefore offering further limited long-term antagonism between bacteriophages and macrophages. Decreases in biofilm production as associated with phage resistance is also favorable outcome as bacteria typically become easier to clear by the immune system and antibiotics [23, 48]. It is therefore valuable to understand the short and longer-term interactions between bacteria, bacteriophage and the immune system; short-term dynamics will determine infection establishment and exacerbation, while longer-term dynamics will determine if the infection can be resolved via single or joint action of the bacteriophage and immune components. This is especially important considering evolutionary changes can increase or decrease a bacteria’s relative susceptibility to antibiotics, different immune components, or other bacteriophages. Future work should aim to characterize how interactions between the immune system and bacteriophage and infecting bacteria change over the course of phage therapy cases.

## Supplementary Material

Supplementary-Material_wrag116

## Data Availability

R code and data are deposited on GitHub: https://github.com/mcastledine96/Macrophages_2025
