# Supplementary material for: Short-term antagonism between bacteriophages and macrophages decreases with bacteria-phage coevolution
Source: ISME J. 2026 May 8;20(1):wrag116. doi: 10.1093/ismejo/wrag116 (PMC13222528; doi:10.1093/ismejo/wrag116)
Supplement: Supplementary-Material_wrag116 [file supplementary-material_wrag116.zip › supplementary_figures_wrag116.docx]

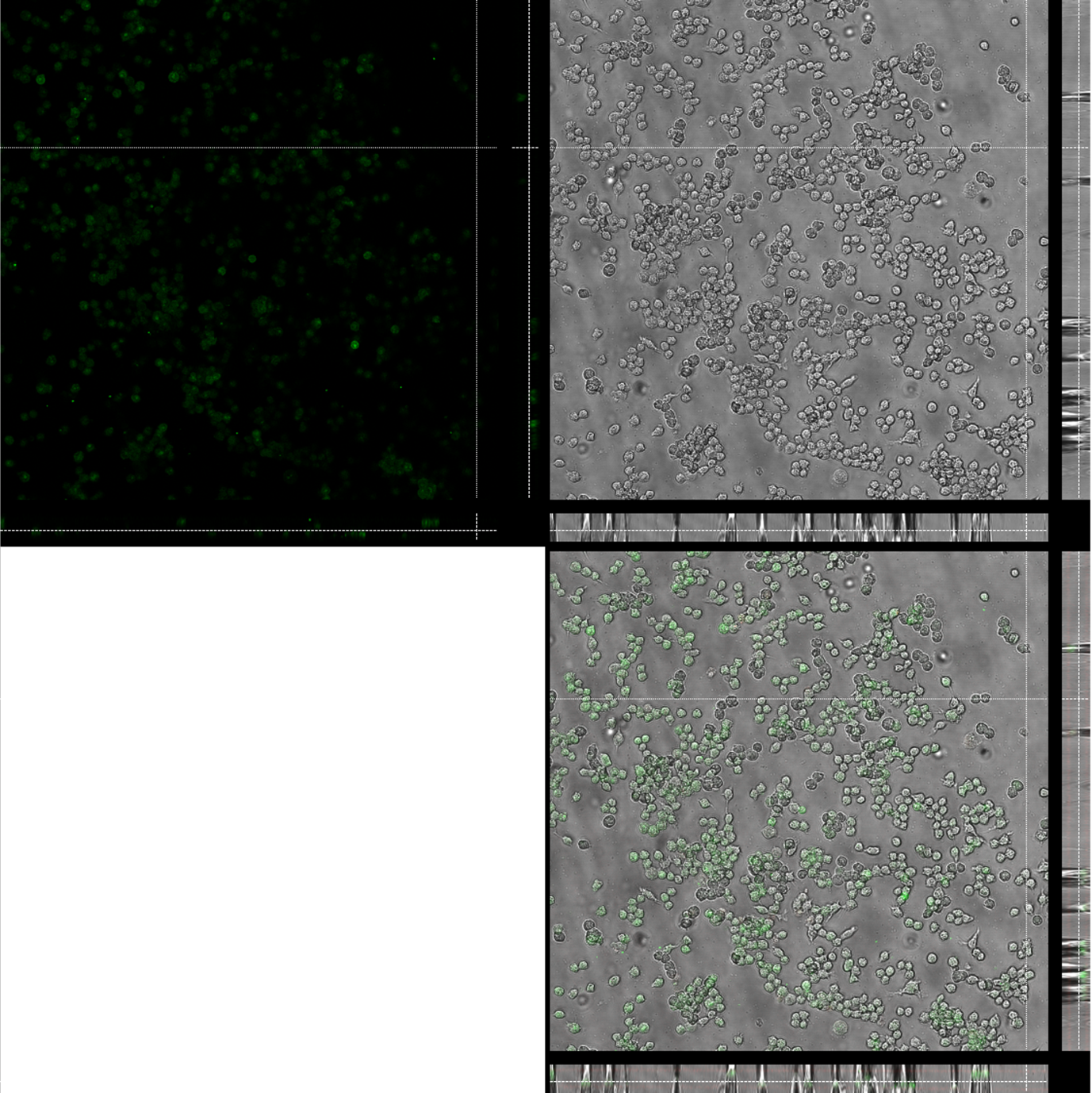


Figure S1. Presence of intracellular *P. aeruginosa* CN573 after 1-2 hours incubating with macrophages. Some free bacterial cells are also visible fluorescing green. The top left panel displays the fluorescence channel, where bacterial signal is visualized in green (excitation wavelength 488 nm, emission wavelength 493–547 nm). The top right panel shows the corresponding transmitted light (brightfield) image of the same field, highlighting macrophage morphology and cell distribution. The lower right panel presents the merged image, demonstrating the intracellular localization of bacteria within macrophages. Orthogonal views (x–z and y–z projections) are shown along the top and right margins, confirming that the fluorescent bacterial signal resides within the cellular boundaries rather than on the surface.


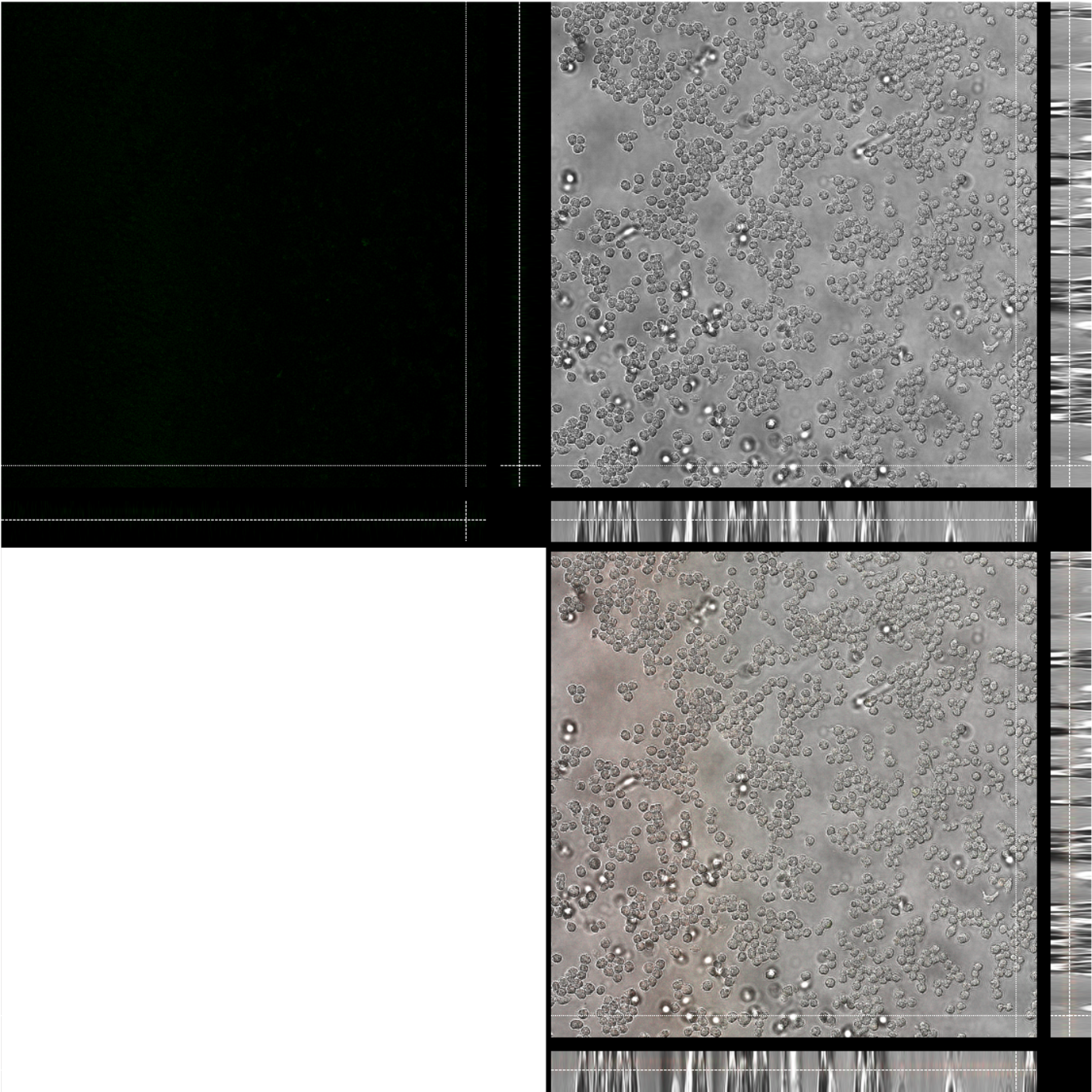


Figure S2. Macrophage-only controls indicating the absence of florescence in the absence of *P. aeruginosa*. The top left panel displays the fluorescence channel, (excitation wavelength 488 nm, emission wavelength 493–547 nm). The top right panel shows the corresponding transmitted light (brightfield) image of the same field. The lower right panel presents the merged image. Out of focus cells occurred in this image due to some cells adhering to the opposite side of the cover slip.


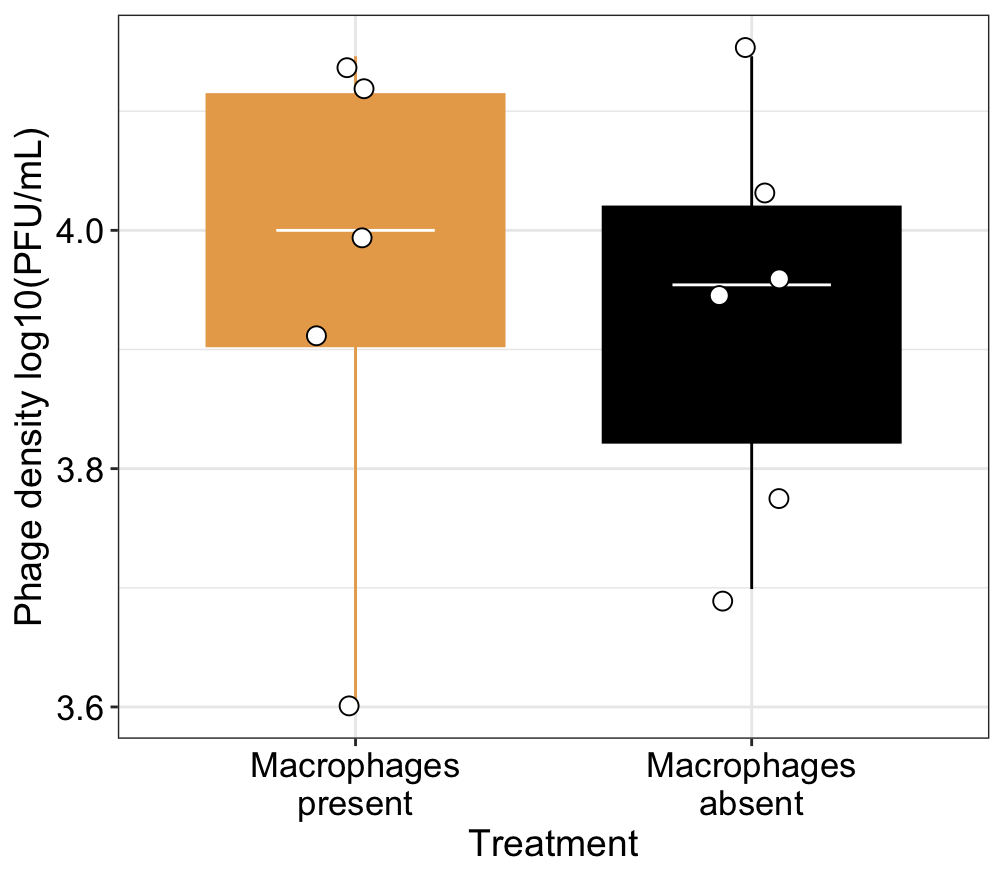


Figure S3. Phage density (log10(PFU/mL)) following culturing with and about macrophages present. Tops and bottoms of the bars represent the 75th and 25th percentiles of the data, the middle lines are the medians, and the whiskers extend from their respective hinge to the smallest or largest value no further than 1.5* interquartile range.


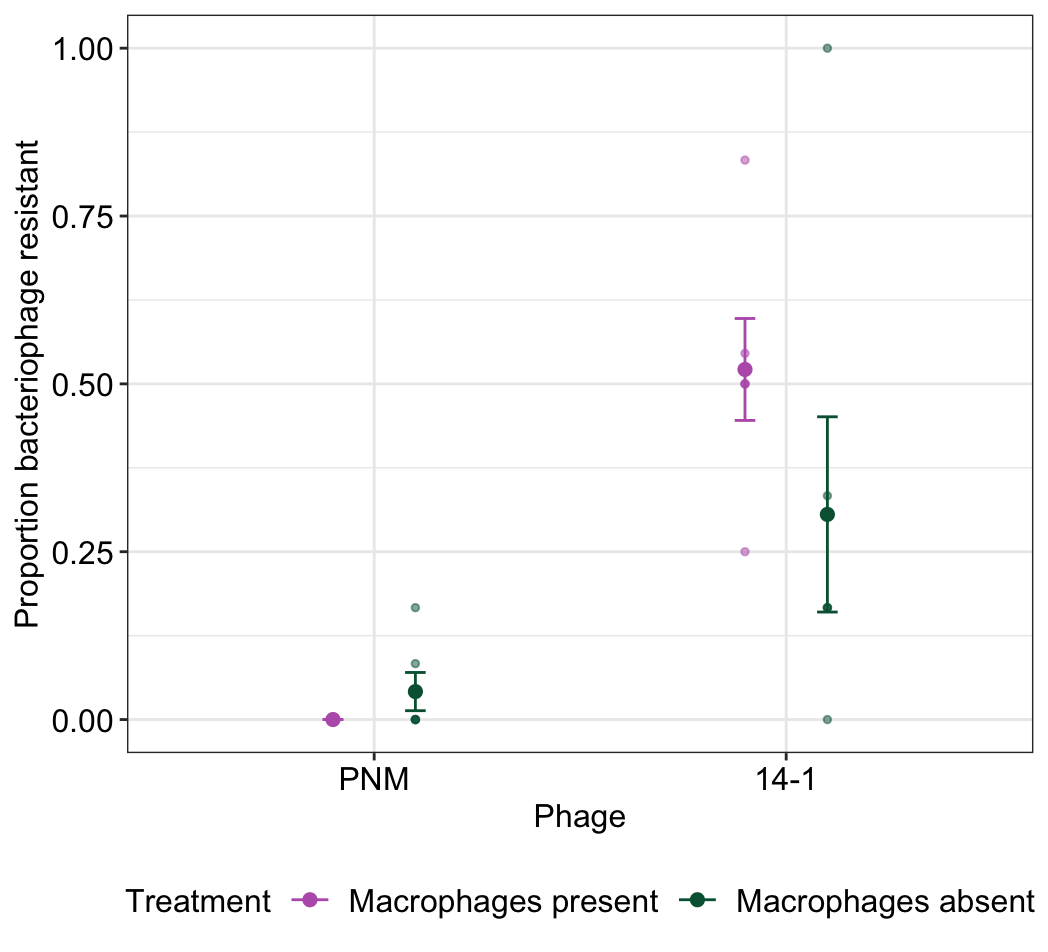


Figure S4. The proportion of isolates within each treatment replicate that were resistant to bacteriophages PNM and 14-1 at day 6 of experimental evolution. Small points indicate individual treatment replicates while larger points with bars indicate means with standard error.


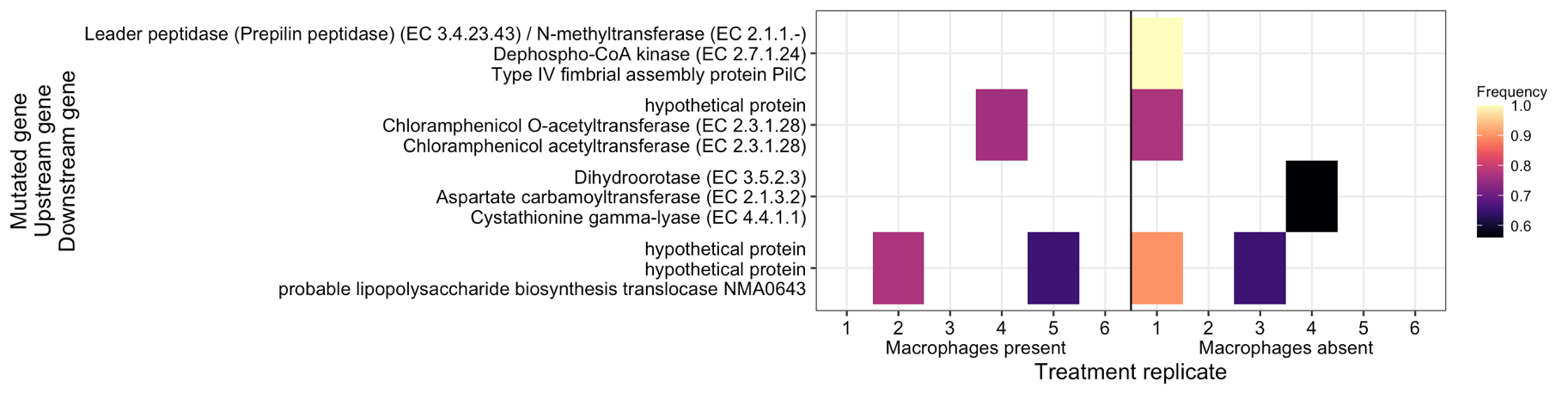


Figure S5. Mutations identified in bacteria populations evolved in the presence of bacteriophages and the presence and absence of macrophages. The mutated gene is presented on the x-axis alongside the upstream and downstream genetic region. Tiles indicate the frequency of the mutation within the population.


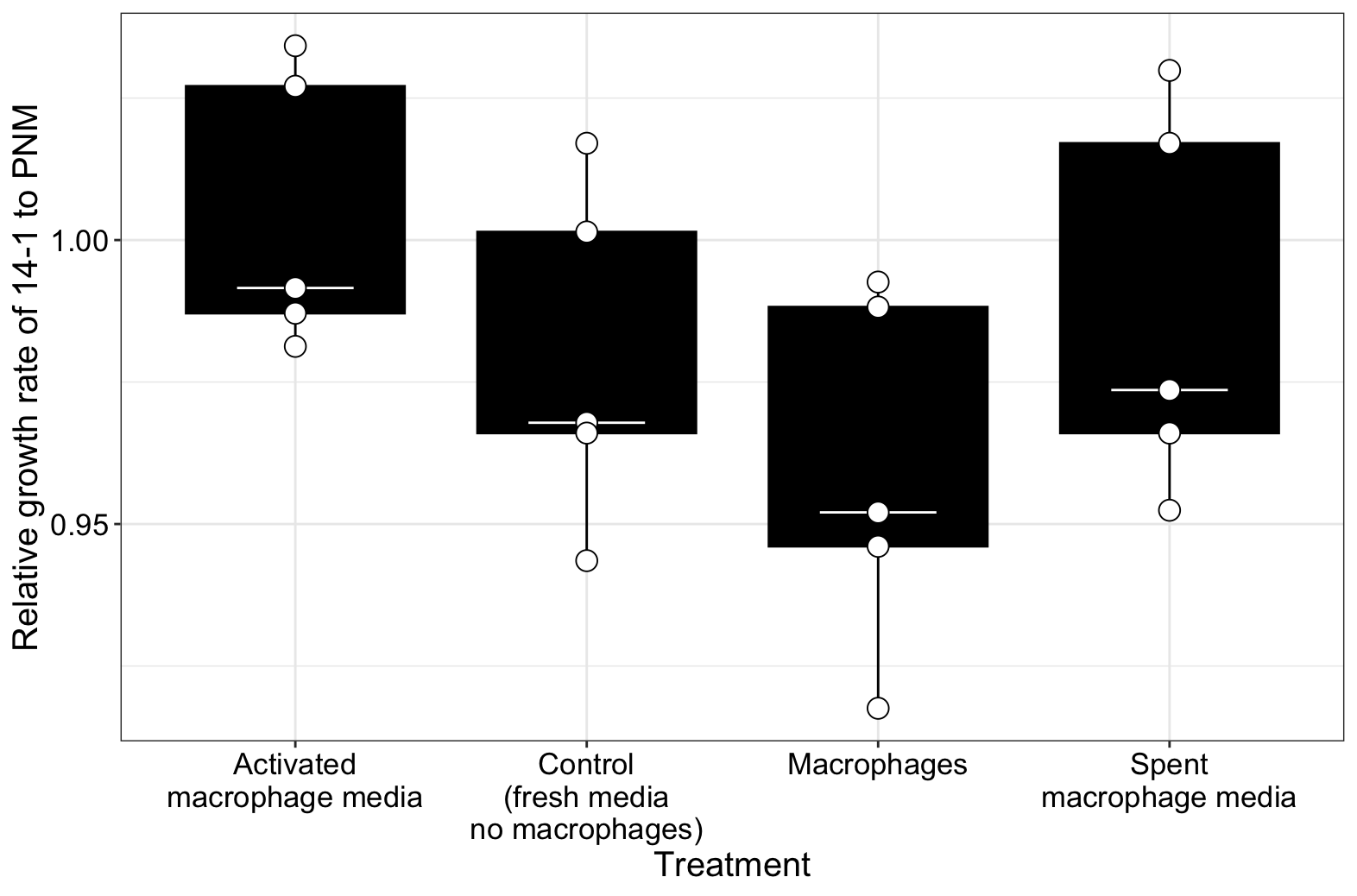


Figure S6. The relative fitness of 14-1 to PNM in different media and macrophage treatments. Points indicate individual treatment replicates. Tops and bottoms of the bars represent the 75th and 25th percentiles of the data, the middle lines are the medians, and the whiskers extend from their respective hinge to the smallest or largest value no further than 1.5* interquartile range.
