# Supplementary material for: Short-term antagonism between bacteriophages and macrophages decreases with bacteria-phage coevolution
Source: ISME J. 2026 May 8;20(1):wrag116. doi: 10.1093/ismejo/wrag116 (PMC13222528; doi:10.1093/ismejo/wrag116)
Supplement: Supplementary-Material_wrag116 [file supplementary-material_wrag116.zip › supplementary_file1_wrag116.docx]

Supplementary File 1. Summary of mutations found in bacteria populations evolved in the absence (BP / N) or presence (BMP / Y) of macrophages and bacteriophages. Nonsyn refers to non-synonymous mutations.

| Sample | Macrophages | Position | Score | Frequency | Type | Reference | Variant | Function | Upstream_feature | Downstream_feature | SNP type |
| --- | --- | --- | --- | --- | --- | --- | --- | --- | --- | --- | --- |
| BP1 | N | 504,704 | 100,699.0 | 0.90 | Nonsyn | agt | Cgt | hypothetical protein | hypothetical protein | probable lipopolysaccharide biosynthesis translocase NMA0643 | missense_variant |
| BMP2 | Y | 505,106 | 74,066.9 | 0.77 | Nonsyn | ttt | Gtt | hypothetical protein | hypothetical protein | probable lipopolysaccharide biosynthesis translocase NMA0643 | missense_variant |
| BP3 | N | 505,394 | 30,368.6 | 0.65 | Deletion | ggaggggggcat | GGAGGGGGCat | hypothetical protein | hypothetical protein | probable lipopolysaccharide biosynthesis translocase NMA0643 | frameshift_variant |
| BMP5 | Y | 505,394 | 41,616.8 | 0.65 | Deletion | ggaggggggcat | GGAGGGGGCat | hypothetical protein | hypothetical protein | probable lipopolysaccharide biosynthesis translocase NMA0643 | frameshift_variant |
| BP4 | N | 276,775 | 15,460.0 | 0.56 | Nonsyn | tac | Gac | Dihydroorotase (EC 3.5.2.3) | Aspartate carbamoyltransferase (EC 2.1.3.2) | Cystathionine gamma-lyase (EC 4.4.1.1) | missense_variant |
| BMP4 | Y | 843,757 | 78,036.7 | 0.76 | Nonsyn | gac | gaG | hypothetical protein | Chloramphenicol O-acetyltransferase (EC 2.3.1.28) | Chloramphenicol acetyltransferase (EC 2.3.1.28) | missense_variant |
| BP1 | N | 844,229 | 60,677.8 | 0.77 | Nonsyn | gtg | Ttg | hypothetical protein | Chloramphenicol O-acetyltransferase (EC 2.3.1.28) | Chloramphenicol acetyltransferase (EC 2.3.1.28) | missense_variant |
| BP1 | N | 114,112 | 119,715.0 | 1.00 | Nonsyn | tgg | tAg | Leader peptidase (Prepilin peptidase) (EC 3.4.23.43) / N-methyltransferase (EC 2.1.1.-) | Dephospho-CoA kinase (EC 2.7.1.24) | Type IV fimbrial assembly protein PilC | stop_gained |
