# Supplementary material for: Short-term antagonism between bacteriophages and macrophages decreases with bacteria-phage coevolution
Source: ISME J. 2026 May 8;20(1):wrag116. doi: 10.1093/ismejo/wrag116 (PMC13222528; doi:10.1093/ismejo/wrag116)
Supplement: Supplementary-Material_wrag116 [file supplementary-material_wrag116.zip › supplementary_tables_wrag116.docx]

Table S1. Anti-phage defences identified in the reference genome of *Pseudomonas aeruginosa* P573 (CN573-PSE143).

| System | Target | HMM accession | HMM name | Protein | Coverage | Start | End | Strand |
| --- | --- | --- | --- | --- | --- | --- | --- | --- |
| RM_type_II | scaffold1.1_50 | PDLC03420 | REase_II_00022 | REase_II | 0.984 | 61912 | 62862 | - |
| RM_type_II | scaffold1.1_51 | PDLC03227 | MTase_II_00013 | MTase_II | 0.984 | 62859 | 64337 | - |
| CRISPR_array | CRISPR001 |  |  | CRISPR_array |  | 963351 | 964758 | - |
| CRISPR_array | CRISPR002 |  |  | CRISPR_array |  | 37803 | 38550 | + |
| CRISPR_array | CRISPR003 |  |  | CRISPR_array |  | 47074 | 48722 | - |
| cas_type_I-F1 | scaffold12.1_26 | PDLC01352 | cd09739 | Cas6f | 0.989 | 38674 | 39237 | - |
| cas_type_I-F1 | scaffold12.1_27 | PDLC01629 | pfam09615 | Cas7f | 0.959 | 39241 | 40269 | - |
| cas_type_I-F1 | scaffold12.1_28 | PDLC01628 | pfam09614 | Cas5f | 0.930 | 40280 | 41263 | - |
| cas_type_I-F1 | scaffold12.1_29 | PDLC01348 | cd09735 | Cas8f | 0.848 | 41250 | 42554 | - |
| cas_type_I-F1 | scaffold12.1_30 | PDLC00941 | cas3_yersinia | Cas23f | 0.994 | 42612 | 45842 | - |
| cas_type_I-F1 | scaffold12.1_31 | PDLC00925 | cas1_YPEST | Cas1f | 0.942 | 45839 | 46813 | - |
| gabija | scaffold2.1_80 | PDLC00063 | GajA_00003 | GajA | 0.959 | 81425 | 83491 | + |
| gabija | scaffold2.1_81 | PDLC00113 | GajB_00011 | GajB | 0.990 | 83484 | 85217 | + |
| PD-T4-6 | scaffold5.1_122 | PD-T4-6_WP_036366335.1 | PD-T4-6_WP_036366335.1 | PD-T4-6 | 0.824 | 131480 | 132469 | + |
| PifA | scaffold6.1_287 | PDLC02361 | PifA | PifA | 0.961 | 327610 | 329751 | - |

Table S2. Multiple pairwise comparisons comparing bacterial densities across four treatments where bacteriophages and macrophages were present (“Both” when together) and absent (“Bacteria”) through days one to six of experimental evolution. P-values adjusted using the Tukey method of comparing a family of four estimates.

| Contrast | Day | Estimate | t-ratio | p-value |
| --- | --- | --- | --- | --- |
| Bacteria - Macrophages | 1 | 0.030 | 0.058 | 1 |
| Bacteria - Bacteriophages | 1 | 4.376 | 8.528 | <0.001 |
| Bacteria - Both | 1 | 3.368 | 6.565 | <0.001 |
| Macrophages - Bacteriophages | 1 | 4.346 | 8.470 | <0.001 |
| Macrophages - Both | 1 | 3.339 | 6.507 | <0.001 |
| Bacteriophages - Both | 1 | -1.007 | -1.963 | 0.209 |
| Bacteria - Macrophages | 2 | 0.630 | 1.228 | 0.611 |
| Bacteria - Bacteriophages | 2 | 5.259 | 10.249 | <0.001 |
| Bacteria - Both | 2 | 2.774 | 5.406 | <0.001 |
| Macrophages - Bacteriophages | 2 | 4.629 | 9.021 | <0.001 |
| Macrophages - Both | 2 | 2.144 | 4.178 | <0.001 |
| Bacteriophages - Both | 2 | -2.485 | -4.842 | <0.001 |
| Bacteria - Macrophages | 3 | 0.688 | 1.340 | 0.54 |
| Bacteria - Bacteriophages | 3 | 2.137 | 4.164 | <0.001 |
| Bacteria - Both | 3 | 1.604 | 3.126 | 0.012 |
| Macrophages - Bacteriophages | 3 | 1.449 | 2.824 | 0.029 |
| Macrophages - Both | 3 | 0.916 | 1.786 | 0.286 |
| Bacteriophages - Both | 3 | -0.533 | -1.038 | 0.728 |
| Bacteria - Macrophages | 4 | 1.592 | 2.668 | 0.043 |
| Bacteria - Bacteriophages | 4 | 1.362 | 2.531 | 0.061 |
| Bacteria - Both | 4 | 2.395 | 4.450 | <0.001 |
| Macrophages - Bacteriophages | 4 | -0.230 | -0.400 | 0.978 |
| Macrophages - Both | 4 | 0.803 | 1.399 | 0.503 |
| Bacteriophages - Both | 4 | 1.033 | 2.013 | 0.19 |
| Bacteria - Macrophages | 5 | 0.896 | 1.319 | 0.553 |
| Bacteria - Bacteriophages | 5 | 2.720 | 4.740 | <0.001 |
| Bacteria - Both | 5 | 3.208 | 5.590 | <0.001 |
| Macrophages - Bacteriophages | 5 | 1.824 | 2.900 | 0.023 |
| Macrophages - Both | 5 | 2.312 | 3.676 | 0.002 |
| Bacteriophages - Both | 5 | 0.488 | 0.951 | 0.777 |
| Bacteria - Macrophages | 6 | 0.878 | 1.292 | 0.57 |
| Bacteria - Bacteriophages | 6 | 1.894 | 3.300 | 0.007 |
| Bacteria - Both | 6 | 2.820 | 4.913 | <0.001 |
| Macrophages - Bacteriophages | 6 | 1.016 | 1.615 | 0.374 |
| Macrophages - Both | 6 | 1.942 | 3.088 | 0.014 |
| Bacteriophages - Both | 6 | 0.926 | 1.805 | 0.277 |

Table S3. Multiple pairwise comparisons comparing bacteriophage density through time (days 1-6). P-values adjusted using the Tukey method of comparing a family of six estimates.

| Contrast | Estimate | t-ratio | p-value |
| --- | --- | --- | --- |
| 1 - 2 | 0.276 | 1.614 | 0.593 |
| 1 - 3 | 0.317 | 1.897 | 0.415 |
| 1 - 4 | 0.216 | 1.292 | 0.788 |
| 1 - 5 | 0.436 | 2.612 | 0.112 |
| 1 - 6 | 0.812 | 4.862 | <0.001 |
| 2 - 3 | 0.041 | 0.237 | 1 |
| 2 - 4 | -0.061 | -0.354 | 0.999 |
| 2 - 5 | 0.160 | 0.934 | 0.936 |
| 2 - 6 | 0.536 | 3.130 | 0.032 |
| 3 - 4 | -0.101 | -0.605 | 0.99 |
| 3 - 5 | 0.119 | 0.714 | 0.979 |
| 3 - 6 | 0.495 | 2.965 | 0.049 |
| 4 - 5 | 0.220 | 1.320 | 0.773 |
| 4 - 6 | 0.596 | 3.570 | 0.009 |
| 5 - 6 | 0.376 | 2.251 | 0.232 |

Table S4. Multiple pairwise comparisons comparing bacteriophage growth rates (averaged over 14-1 and PNM as non-significant in the model) in treatments in which the environment was manipulated. The treatment environment included a ‘no macrophage control’ (fresh growth media, no macrophages), ‘macrophages’ (fresh media and unstimulated macrophages), ‘activated media’ (media from macrophages stimulated by heat-killed bacteria) and ‘spent media’ (media isolated from unstimulated macrophage growth). P-values adjusted using the Tukey method of comparing a family of four estimates.

| Contrast | Estimate | t-ratio | p-value |
| --- | --- | --- | --- |
| No Macrophage Control - Activated Media | -0.112 | -0.866 | 0.822 |
| No Macrophage Control - Macrophages | 1.527 | 11.770 | <0.001 |
| No Macrophage Control – Spent Media | -0.019 | -0.146 | 0.999 |
| Activated Media - Macrophages | 1.639 | 12.636 | <0.001 |
| Activated Media – Spent Media | 0.093 | 0.720 | 0.888 |
| Macrophages – Spent Media | -1.546 | -11.916 | <0.001 |

Table S5. Multiple pairwise comparisons comparing biofilm production of bacteria from different evolutionary backgrounds. P-values adjusted using the Tukey method of comparing a family of five estimates.

| Contrast | Estimate | t-ratio | p-value |
| --- | --- | --- | --- |
| Ancestor - (Bacteria-only) | 0.043 | 0.396 | 0.994 |
| Ancestor - Bacteriophage | -0.126 | -1.241 | 0.729 |
| Ancestor - (Bacteriophage+Macrophage) | 0.162 | 1.606 | 0.516 |
| Ancestor - Macrophage | 0.139 | 1.285 | 0.704 |
| (Bacteria-only) - Bacteriophage | -0.168 | -1.665 | 0.482 |
| (Bacteria-only) - (Bacteriophage+Macrophage) | 0.120 | 1.183 | 0.761 |
| (Bacteria-only) - Macrophage | 0.096 | 0.888 | 0.897 |
| Bacteriophage - (Bacteriophage+Macrophage) | 0.288 | 3.767 | 0.014 |
| Bacteriophage - Macrophage | 0.264 | 2.615 | 0.117 |
| (Bacteriophage+Macrophage) - Macrophage | -0.024 | -0.233 | 0.999 |

Table S6. Multiple pairwise comparisons comparing cytokine production (TNF-α) of macrophages exposed to bacteria from different evolutionary backgrounds. The macrophage-only control refers to the cytokines produced by macrophages not exposed to bacteria. P-values adjusted using the Tukey method of comparing a family of six estimates.

| Contrast | Estimate | t-ratio | p-value |
| --- | --- | --- | --- |
| Ancestor - (Bacteria-only) | 0.256 | 0.864 | 0.950 |
| Ancestor - Bacteriophage | 0.306 | 1.196 | 0.833 |
| Ancestor - (Bacteriophage+Macrophage) | 0.463 | 1.809 | 0.485 |
| Ancestor - Macrophage | 0.306 | 1.033 | 0.900 |
| Ancestor - Macrophage Control | 4.060 | 13.726 | <0.001 |
| (Bacteria-only) - Bacteriophage | 0.051 | 0.198 | 1.000 |
| (Bacteria-only) - (Bacteriophage+Macrophage) | 0.208 | 0.811 | 0.962 |
| (Bacteria-only) - Macrophage | 0.050 | 0.169 | 1.000 |
| (Bacteria-only) - Macrophage Control | 3.805 | 12.861 | <0.001 |
| Bacteriophage - (Bacteriophage+Macrophage) | 0.157 | 0.751 | 0.972 |
| Bacteriophage - Macrophage | -0.001 | -0.003 | 1.000 |
| Bacteriophage - Macrophage Control | 3.754 | 14.653 | <0.001 |
| (Bacteriophage+Macrophage) - Macrophage | -0.158 | -0.616 | 0.988 |
| (Bacteriophage+Macrophage) - Macrophage Control | 3.597 | 14.040 | <0.001 |
| Macrophage - Macrophage Control | 3.755 | 12.692 | <0.001 |
